# Morphometric analysis of the greater palatine foramen: a CBCT study in Portugal

**DOI:** 10.1007/s00276-025-03566-3

**Published:** 2025-01-28

**Authors:** Tomás Martins, Vanessa Guedes, Eugénio Martins, Pedro Mesquita

**Affiliations:** 1 Speciality of Prosthodontics and Occlusion at Faculty of Dentistry, Complutense University of Madrid, Madrid, Spain; 2 Private Practice of Dental Medicine, Vila Real, Portugal; 3 Faculty of Dental Medicine, University of Porto, Porto, Portugal

**Keywords:** Greater palatine foramen, Cone beam computed tomography, Maxilla, Hard palate, Oral anatomy

## Abstract

**Purpose:**

The greater palatine foramen (GPF) represents the inferior opening of the greater palatine canal and is located posterolaterally on both sides of the hard palate. The aim of this study is to morphometrically characterise the GPF and to determine its anatomical relationships in a Portuguese population.

**Methods:**

A retrospective study was performed based on the clinical records which included all permanent teeth erupted and a cone beam computed tomography (CBCT) of the entire maxilla. The diameters of the GPF and its distances to posterior nasal spine (PNS), posterior border of the hard palate (PBHP), anterior nasal spine (ANS), midline maxillary suture (MMS) and incisive foramen (IF) were measured. Its position in relation to molars and the shape and direction of opening towards the oral cavity were classified. Global descriptive and comparative analysis were conducted.

**Results:**

*N* = 100. Metric analysis (mean in millimetres): anteroposterior diameter 5.35, lateromedial diameter 2.24, GPF-PNS 16.26, GPF-PBHP 4.83, GPF-ANS 49.94, GPF-MMS 14.74, GPF-IF 35.18. Position in relation to molars (%): 0% anterior to 2nd molar, 3% opposite 2nd molar, 15% between 2nd and 3rd molars, 77% opposite 3rd molar, 5% distal to 3rd molar. Shape (%): oval 71%, slit 26%, round 1%, other 2%. Metric variables were higher in males than in females (95% CI). There were no differences between genders for categorical variables or between sides for any variable (95% CI).

**Conclusion:**

The most common shape of the GPF is oval and its most common position is opposite the 3rd molar.

## Introduction

The pterygopalatine fossa (PPF) is an inverted pyramid-shaped space situated in the lateral region of the skull, on the medial side of the infratemporal fossa. The PPF is connected posteriorly to the middle cranial fossa via the round foramen and the pterygoid canal (Vidius canal), superiorly to the orbit through the inferior orbital fissure, medially to the nasal cavity through the sphenopalatine foramen, laterally to the infratemporal fossa through the pterygomaxillary fissure, and inferiorly with the oral cavity through the greater palatine canal (GPC) [1, 2, 8, 24]. 

The GPC, which extends inferiorly from the PPF to the greater palatine foramen (GPF), contains a vasculonervous bundle comprising the palatine artery and the greater and lesser palatine nerves [2, 4, 18, 24, 25].

The hard palate is formed anteriorly by the palatine apophyses of the maxillary bones and posteriorly by the horizontal blades of the palatine bones. As these are paired bones, they meet in the midline and form a continuous bony rim, which constitutes the hard palate [16]. 

The GPF represents the inferior opening of the greater palatine canal and is located posterolaterally on both sides of the hard palate. There is no consensus in the literature regarding the precise location of the GPF. However, the most common location of the GPF is at the level of the third molar [3, 10, 14, 17–18, 22, 23].

Similarly to the GPC, the GPF contains the greater palatine nerve (GPN) and the greater palatine artery (GPA) [14].

The GPN runs anteriorly in the palate to the proximity of the alveolar crest in the area of the incisor teeth, where it communicates with the terminal branches of the nasopalatine nerves [4, 12]. The GPN is responsible for innervating the mucosa of the hard palate, the medial wall of the maxillary sinus and the posterior part of the lateral wall of the nasal cavity [9, 18]. 

The GPA runs in an anterior direction in close proximity to the alveolar crest and subsequently enters the nasal cavity through the incisive foramen, where it anastomoses with the posterior septal branch of the sphenopalatine artery. The diameter of the GPA is greatest when it emerges at the level of the GPF and gradually decreases as it approaches the incisive foramen. Its branches are predominantly located in the area of the premolars, more frequently on the alveolar side than on the hard palate side [12]. The GPA provides vascularisation to the mucosa of the palate and the periodontium of the maxillary posterior teeth [14, 19]. 

As a reference site for the GPN and the GPA, a precise knowledge of the location of the GPF is relevant to avoid iatrogenic neurovascular injuries in dental procedures, including anaesthetic techniques, upper molar extractions, the placement of orthodontic mini-implants, cystic or tumour pathology surgeries, and the harvesting of connective tissue grafts. [9, 12, 18] Although existing studies provide a comprehensive overview of the GPF’s anatomy, it is important to consider potential anatomical variations and their implications, as these can significantly influence the outcomes of dental anesthesia and surgical planning. Such inadvertent damage can result in paresthesia, difficult-to-control haemorrhages or necrosis of palate tissues. The most common iatrogenic injury to the GPA occurs in periodontal surgery to harvest subepithelial connective tissue grafts [4, 10, 12, 18].

The GPN block is a common and straightforward procedure that involves an intraoral approach to the nerve. It is recommended for surgical procedures involving the upper molars, maxillary sinus, or nasal region. However, knowledge of the anatomical variations in the position and morphology of the GPF is required. [3, 9–10, 17, 19, 20, 21, 24] Malamed et al. (2017) located the GPF by palpating it midway between the gingival margin and the midline of the palate, approximately opposite to the distal face of the maxillary second molar [15]. 

Various studies have been carried out to analyse the GPF, both in terms of its location and shape, as well as its anatomical relationships. The most commonly used methods are human skeletal observation and cone beam computed tomography (CBCT) scans [1, 8, 10, 14]. 

CBCT is a three-dimensional x-ray imaging technique that overcomes the limitations of traditional two-dimensional dental imaging and allows accurate imaging of details of the maxillofacial bone structures and surrounding soft tissues [11]. 

The fact that we could not find any published studies analysing the GPF in a Portuguese population was the main reason for carrying out this study. The aim of this study is to morphometrically characterise the GPF, determine its anatomical relationships, as well as to define its location based on CBCT scans of a Portuguese sample.

## Materials and methods

This study was based on an analysis of CBCT scans of individuals who had undergone an examination of the entire maxilla. The records were obtained from the archive of a private dental clinic, after the requisite authorisation, between February and May, 2024. This study was approved by the Health Ethics Committee of the Faculty of Dental Medicine, University of Porto, Porto, Portugal, and by the Data Protection Unit from the same University. The CBCT images were anonymously consulted merely for research purposes with previous patients’ consent.

The CBCT images were obtained using the equipment Planmeca ProMid^®^ 3D using Amorphous Silicon Flat Panel sensor with the following specifications: 90 kV, 8 mA, 40 s exposure time and 0,4 mm voxel dimension. The operator and the equipment operation protocol remained the same throughout the image acquisition.

The image analysis was done using the software Planmeca Romexis^®^, Helsinki, Finland.

To estimate the sample size for each group, data from measurements obtained from the first 10 individuals were used. The sample size was calculated using the Statulator online calculator (Dhand & Khatkar, 2014) [6], considering a confidence level of 95%, a maximum margin of error corresponding to 10% of the mean and a sample standard deviation of 10 patients.

Of the variables included in the study, the one that maximizes the sample size was considered. Under these conditions, a sample of 25 individuals in each group (25 men and 25 women) allowed us to estimate the average GPF measurements in each group with a confidence level of 95%.

From a data base of 2000 CBCT scans that met the study ethical requirements, the images were consulted sequentially from the most recent to the oldest and meeting the inclusion and exclusion criteria, until a sample of 25 females and 25 males was achieved. To achieve this sample 1256 scans were consulted.

The study included individuals of both genders, aged 18 years old or over, who had undergone an examination that encompassed the entire maxilla. Each hemi-arch had to comprise a central incisor, a lateral incisor, a canine, a first and a second premolar, and a first, a second and a third molar, with all upper teeth fully erupted. All images with a voxel size of 0.4 mm or less were included.

The patient records were excluded based on the following criteria: the ones which did not encompass the entire maxilla, which were not adequately documented regarding to the patient’s research data, cases involved history of trauma, cases which had a history of craniofacial or orthognathic surgery, which exhibited signs of pathological bone resorption in the jaws, or cases with absence of ossification of the posterior mandibular bone. Dental implants or artefacts in the areas of interest were also excluded. Additionally, cases with poor image quality were not included.

The assessment of the CBCT scans was performed by a Doctor of Dental Surgery with specific training in analysis of CBCT and 3 years of clinical experience.

To avoid errors, the operator applied the following objective and detailed 6-step protocol 10 times in different CBCT scans before carrying out the investigation.

**Fig. 1:**
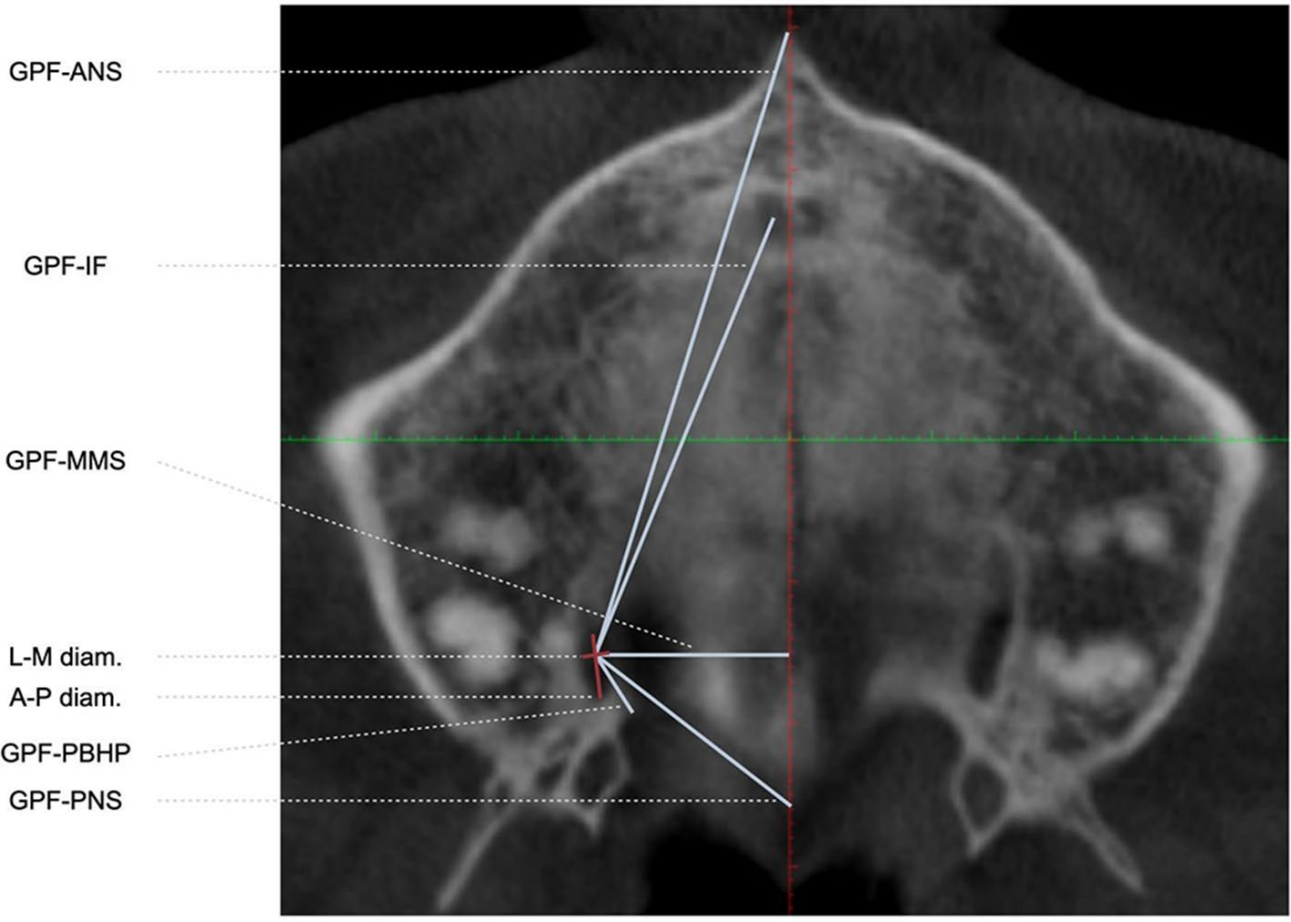
Illustration of step 3 in a cross section of CBCT in the hard palate

**Fig. 2:**
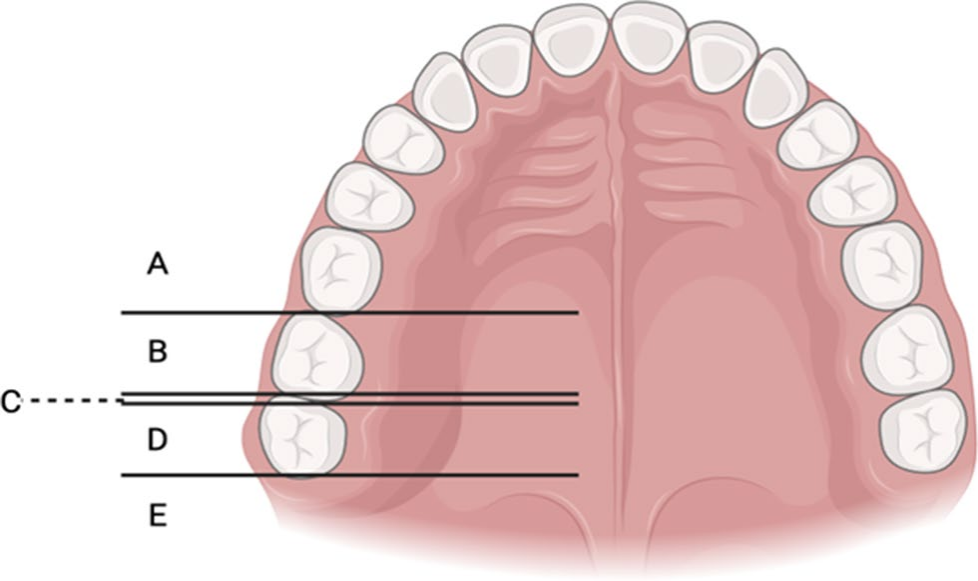
Illustration of the classification of the position of GPF in relation to molars (step 4)

1. Orientation of the 3D image on the three planes. Axial section: along the palatine apophyses at a midpoint between the upper and lower bone plates in the maxilla; coronal section: the midline is coincident with the ANS and PNS; sagittal section: at the level of the right and left GPF, respectively.
2. Location of the centres of the right and left GPF, in accordance with the methodology described by Tomaszewska et al. (2014) [23]. The centre of the right and left GPF was identified as the point of intersection between the two straight lines formed by the largest anteroposterior and lateromedial dimensions of each GPF.
3. Measurement of the distance, in millimetres, from the centre of the right and left GPF (Fig. 1):

- The distance between the GPF and the posterior nasal spine (GPF-PNS);
- The shortest distance between the GPF and the posterior border of the hard palate (GPF - PBHP);
- The distance between the GPF and the anterior nasal spine (GPF - ANS);
- The shortest perpendicular distance between the GPF and the midline maxillary suture (GPF - MMS);
- The distance between the GPF and the centre of the right or left incisive foramen, respectively (GPF - IF);
- The greatest anteroposterior distance of the GPF - Anteroposterior diameter of the GPF;
- The greatest lateromedial distance of the GPF - GPF lateromedial diameter.

4. Classification of the position of the right and left GPF in relation to the maxillary second and third molars (Fig. 2), considering the lines perpendicular to the midpalatal suture passing through the centre of the GPF - proposed by Tomaszewska et al. (2014) [23]:

A. GPF positioned mesial to the second maxillary molar (2MM) (mesial/anterior to the mesial surface of the 2MM);
B. GPF positioned opposite the 2MM (between the mesial and distal faces of the 2MM);
C. GPF positioned between the 2MM and the third maxillary molar (3MM) (interproximal, at the level of the contact surface between the 2MM and the 3MM);
D. GPF positioned opposite the 3MM (between the mesial and distal surfaces of the 3MM);
E. GPF positioned distal to the 3MM (distal/posterior to the distal face of the 3MM).

5. Observational classification of the shape of the right and left GPF: (A) Oval/ovoid; (B) Round; (C) Slit; (D) Other shape.
6. Direction of opening of the right and left GPF into the oral cavity in relation to the sagittal plane: (A) Anterior; (B) Inferior-Anterior-Medial (IAM); (C) Inferior-Anterior-Lateral (IAL); (D) Vertical.

Data collection was repeated by the same operator for 30 assessments 15 days after the primary data collection (15 left and 15 right) to calculate the measurement error.

### Statistical analysis

The statistical analysis was performed using the Statistical Package for the Social Sciences (SPSS) software, version 29 for Windows (IBM Corp., 2022).

A sample of 30 evaluations of 15 patients was selected for the measurement of the error (15 evaluations of the right side and 15 evaluations of the left side). The measurement error of the metric variables was assessed using Student’s T-test for paired samples and the Intraclass Correlation Coefficient (ICC).

For the categorical variables the concordance rate and the Cohen’s Kappa Coefficient was applied.

The metric variables were characterised using the mean and the corresponding 95% confidence interval (95% CI). The categorical variables were characterised using absolute (n) and relative (%) frequencies and 95% confidence intervals (95% CI) for relative frequencies.

The values obtained from the sample in this study were compared with the reference values (RV) taken from the article by Kim et al. (2023) [12], as presented in Tables 3 and 4.

The independent samples T-test was employed to evaluate the comparison of metric variables according to gender (female versus male) and side (right versus left). Conversely, the Fisher’s Exact Test was utilised for the comparison of categorical variables based on gender and position.

A significance level of 5 per cent was employed, whereby differences were deemed statistically significant when the associated significance value was less than 0.05 (*p* < 0.05).

## Results

The results were estimated with a 95% confidence interval (CI) based on a sample of 100 GPF.

### Measurement error

The results of Student’s T-tests for paired samples show that there are no statistically significant differences (*p* > 0.05) between the means of the first measurement and the means of the second measurement. The Intraclass Correlation Coefficient (ICC) values were greater than 0.90 in all variables, indicating excellent consistency between the first measurement and repetition (Table 1). Taken together, these results guarantee excellent reliability of the measurements performed and confirm the absence of measurement error of the metric variables.

Regarding the categorical variables (position, shape and direction of the opening of the GPF), perfect concordance was recorded between the initial measurements and the reassessments (concordance = 100%, Cohen’s Kappa = 1.000), indicating the absence of measurement error in these variables (Table 1).

### Sample

The sample consisted of 25 females (50%) and 25 males (50%), with an age range between 18 and 56 years of age (the same age range in both groups). The mean age was 29.2 years old (SD = 9.8). No significant differences were observed between the mean age of the female and male participants (29.2 years old, SD = 10.2 and 29.2 years old, SD = 9.6, respectively; *p* = 1.000; Table 2).

### GPF morphometric analysis

#### Overall characterization and comparison with reference values

For the overall characterization of the measurements assessed, the measurements of the female and male patients and the right and left sides (*N* = 100) were considered together, allowing for a comprehensive data overview. The results are presented in Tables 3 and 4. The mean GPF-PNS distance in the sample was 16.26 (95% CI = 15.96–16.55), which was not significantly different from the reference average: 17.21 (95% CI = 16.34–18.09). Additionally, no significant discrepancies were observed between the sample and the reference mean for the GPF-MMS distance (sample: 14.74, 95% CI = 14.47-15.00; reference: 15.22, 95% CI = 15.00-15.43) and in the anteroposterior diameter (sample: 5.35, 95% CI = 5.14–5.56; reference: 5.34, 95% CI = 4.99–5.68) (Table 3). The sample mean is significantly higher than the reference mean for the GPF-PBHP distance (sample: 4.83, 95% CI = 4.60–5.05; reference: 2.56, 95% CI = 1.90–3.22) and the GPF-ANS distance (sample: 49.94, 95% CI = 49.32–50.55; reference: 46.24, 95% CI = 44.30-48.18) (Table 3). On the other hand, the sample mean is significantly lower than the reference mean in the GPF-IF distance (sample: 35.18, 95% CI = 34.64–35.72; reference: 37.32, 95% CI = 36.19–38.45) and in the lateromedial diameter (sample: 2.24, 95% CI = 2.15–2.34; reference: 2.77, 95% CI = 2.58–2.96) (Table 3).

Regarding the position of the GPF (Table 4), none were in position A, 3.0% (95% CI = 0.9–7.8) were in position B, 15.0% (95% CI = 9.0–23.0) were in position C, 77.0% (95% CI = 68.1–84.4) were in position D and 5.0% (95% CI = 1.9–10.6) were in position E. These percentages do not differ significantly from the reference values (with the exception of position A).

As for shape (Table 4), the sample was predominantly oval/ovoid (71.0%, 95% CI = 61.6–79.2), followed by slit-shaped (26.0%, 95% CI = 18.2–35.2) and round-shaped (1.0%, 95% CI = 0.1–4.6). These percentages do not differ significantly from the reference values (with the exception of the “other” shape − 2.0% (95% CI = 0.4–6.3)).

Of the 100 GPF evaluated, 89.0% (95% CI = 81.8–94.0) had an IAM opening direction and 11.0% (95% CI = 6.0-18.2) had an IAL position (Table 4). The percentage of cases with IAM opening is significantly higher in the sample than the reference values (54.5%, 95% CI = 40.4–67.9) and the percentage of cases with IAL opening does not differ significantly from the reference values (14.4%, 95% CI = 4.9–35.4) (Table 4).

#### Morphometric analysis of GPF by gender

Tables 5 and 6 show the results of the characterization of the GPF assessments in female and male patients. With regard to the metric variables (Table 5), the measurements of the male patients are all significantly higher than those of the female patients (*p* < 0.05) (Table 5).

There were no statistically significant differences between female and male patients with regard to the position (*p* = 0.521), shape (*p* = 0.103) or opening direction of the GPF (*p* = 1.000) (Table 6).

### Morphometric analysis of the GPF per side

There were no statistically significant differences (*p* > 0.05) between the GPFs on the right and left sides with regard to the metric variables (Table 7) or with regard to the position, shape or direction of the opening (Table 8).

**Table 1:** Measurement error results (*N* = 30)

Metric variables	Student’s T-test	ICC
Distance GPF-PNS	*p* = 0.814	0.988
Distance GPF-PBHP	*p* = 0.620	0.979
Distance GPF-ANS	*p* = 0.262	0.996
Distance GPF-MMS	*p* = 0.797	0.957
Distance GPF-IF	*p* = 0.300	0.975
Antero-posterior diameter	*p* = 0.348	0.961
Lateromedial diameter	*p* = 0.361	0.905
**Categorical variables**	**Concordance rate**	**Cohen’s Kappa coefficient**
Position	100%	1.000
Shape	100%	1.000
Opening direction	100%	1.000

**Table 2:** Characterization of the sample in terms of age

Age	Female (*N* = 25)	Male (*N* = 25)	Total (*N* = 50)
Minimum - maximum	18–56	18–56	18–56
Mean (standard deviation)	29.2 (10.2)	29.2 (9.6)	29.2 (9.8)
*Student’s T-Test*	*p* = 1.000		

**Table 3:** Global GPF metric analysis and comparison with reference values

Measurements	Sample (*N* = 100)	Reference values ^(1)^	CI 95% interception ^(2)^
	Mean (CI 95%)	Mean (CI 95%)	
Distance GPF-PNS	**16.26** (15.96–16.55)	**17.21** (16.34–18.09)	Yes
Distance GPF-PBHP	**4.83** (4.60–5.05)	**2.56** (1.90–3.22)	No
Distance GPF-ANS	**49.94** (49.32–50.55)	**46.24** (44.30–48.18)	No
Distance GPF-MMS	**14.74** (14.47–15.00)	**15.22** (15.00–15.43)	Yes
Distance GPF-IF	**35.18** (34.64–35.72)	**37.32** (36.19–38.45)	No
Antero-posterior diameter	**5.35** (5.14–5.56)	**5.34** (4.99–5.68)	Yes
Lateromedial diameter	**2.24** (2.15–2.34)	**2.77** (2.58–2.96)	No

**(1)** Kim et al. (2023); **(2)** If the 95% CIs intersect, the sample mean does not differ significantly from the reference mean. If the 95% CIs do not intersect, the sample mean differs significantly from the reference mean

**Table 4:** Global analysis of the position and shape of the GPF and comparison with reference values

Variables	Sample (*N* = 100)	Reference Values ^(1)^	CI 95% interception ^(2)^
	% (CI 95%)	% (CI 95%)	
**Position**			
A	**0.0%**	**3**,**3%** (0,5–20,3)	-
B	**3.0%** (0.9–7.8)	**5**,**0%** (3,2–3,9)	Yes
C	**15.0%** (9.0–23.0)	**19**,**3%** (15,3–24,0)	Yes
D	**77.0%** (68.1–84.4)	**64**,**9%** (58,7–70,7)	Yes
E	**5.0%** (1.9–10.6)	**6**,**0%** (3,7–9,6)	Yes
**Shape**			
Oval/ovoid	**71.0%** (61.6–79.2)	**77.8%** (57.6–90.0)	Yes
Slit	**26.0%** (18.2–35.2)	**8.4%** (2.4–25.8)	Yes
Round	**1.0%** (0.1–4.6)	**9.4%** (3.3–23.8)	Yes
Other	**2.0%** (0.4–6.3)	**35.3%** (14.3–64.0)	No
**Opening direction**			
IAL	**11.0%** (6.0–18.2)	**14.4%** (4.9–35.4)	Yes
IAM	**89.0%** (81.8–94.0)	**54.5%** (40.4–67.9)	No

**(1)** Kim et al. (2023); **(2)** If the 95% CIs intersect, the sample mean does not differ significantly from the reference mean. If the 95% CIs do not intersect, the sample mean differs significantly from the reference mean

**Table 5:** Analysis of metric quantitative variables by gender

Measurements	Female (*N* = 50)		Male (*N* = 50)		Student’s T Test
	Mean	CI 95%	Mean	CI 95%	
Distance GPF-PNS	**15.85**	15.50–16.20	**16.66**	16.20–17.13	*p* = 0.006
Distance GPF-PBHP	**4.59**	4.29–4.90	**5.06**	4.73–5.39	*p* = 0.039
Distance GPF-ANS	**47.94**	47.22–48.66	**51.93**	51.30–52.56	*p* < 0.001
Distance GPF-MMS	**14.28**	13.92–14.64	**15.19**	14.83–15.56	*p* < 0.001
Distance GPF-IF	**33.73**	33.00–34.46	**36.63**	36.05–37.20	*p* < 0.001
Anteroposterior diameter	**5.04**	4.69–5.39	**5.66**	5.45–5.87	*p* = 0.003
Lateromedial diameter	**2.01**	1.89–2.13	**2.48**	2.36–2.59	*p* < 0.001

**Table 6:** Analysis of categorical variables by gender

Variables	Female (*N* = 50)			Male (*N* = 50)			Fisher’s exact test
	*n*	%	CI 95%	*n*	%	CI 95%	
**Position**							
A	0	**0.0%**		0	**0.0%**		*p* = 0.521
B	2	**4.0%**	0.8 − 12.2%	1	**2.0%**	0.2 − 9.0%	
C	10	**20.0%**	10.8 − 32.6%	5	**10.0%**	3.9 − 20.5%	
D	36	**72.0%**	58.6 − 83.0%	41	**82.0%**	69.7 − 90.7%	
E	2	**4.0%**	0.8 − 12.2%	3	**6.0%**	1.7 − 15.2%	
**Shape**							
Oval/ovoid	31	**62.0%**	48.2 − 74.5%	40	**80.0%**	67.4 − 89.2%	*p* = 0.103
Slit	17	**34.0%**	22.1 − 47.7%	9	**18.0%**	9.3 − 30.3%	
Round	1	**2.0%**	0.2 − 9.0%	0	**0.0%**		
Other	1	**2.0%**	0.2 − 9.0%	1	**2.0%**	0.2 − 9.0%	
**Opening Direction**							
IAL	6	**12.0%**	5.2 − 23.1%	5	**10.0%**	3.9 − 20.5%	*p* = 1.000
IAM	44	**88.0%**	76.9 − 94.8%	45	**90.0%**	79.5 − 96.1%	

**Table 7:** Analysis of metric variables, by side

Measurements	Right (*N* = 50)		Left (*N* = 50)		Student’s T test
	Mean	CI 95%	Mean	CI 95%	
Distance GPF-PNS	**16.21**	15.79–16.64	**16.30**	15.87–16.73	*p* = 0.785
Distance GPF-PBHP	**4.67**	4.38–4.96	**4.98**	4.63–5.33	*p* = 0.170
Distance GPF-ANS	**50.07**	49.10–51.04	**49.80**	49.00–50.60	*p* = 0.668
Distance GPF-MMS	**14.80**	14.41–15.19	**14.67**	14.29–15.05	*p* = 0.636
Distance GPF-IF	**35.20**	34.36–36.04	**35.15**	34.44–35.87	*p* = 0.928
Anteroposterior diameter	**5.29**	5.01–5.57	**5.41**	5.09–5.73	*p* = 0.569
Lateromedial diameter	**2.25**	2.12–2.39	**2.23**	2.10–2.37	*p* = 0.814

**Table 8:** Analysis of categorical variables, by side

Variables	Right (*N* = 50)			Left (*N* = 50)			Fisher’s Exact Test
	*n*	%	CI 95%	*n*	%	CI 95%	
**Position**							
A	0	**0.0%**		0	**0.0%**		*p* = 0.754
B	2	**4.0%**	0.8 − 12.2%	1	**2.0%**	0.2 − 9.0%	
C	6	**12.0%**	5.2 − 23.1%	9	**18.0%**	9.3 − 30.3%	
D	40	**80.0%**	67.4 − 89.2%	37	**74.0%**	60.7 − 84.6%	
E	2	**4.0%**	0.8 − 12.2%	3	**6.0%**	1.7 − 15.2%	
**Shape**							
Oval/ovoid	36	**72.0%**	58.6 − 83.0%	35	**70.0%**	56.4 − 81.3%	*p* = 1.000
Slit	13	**26.0%**	15.4 − 39.3%	13	**26.0%**	15.4 − 39.3%	
Round	0	**0.0%**		1	**2.0%**	0.2 − 9.0%	
Other	1	**2.0%**	0.2 − 9.0%	1	**2.0%**	0.2 − 9.0%	
**Opening Direction**							
IAL	7	**14.0%**	6.5 − 25.5%	4	**8.0%**	2.8 − 17.9%	*p* = 0.525
IAM	43	**86.0%**	74.5 − 93.5%	46	**92.0%**	82.1 − 97.2%	

## Discussion

### Overall characterisation and comparison with reference values

In order to compare the data obtained in this study and the existing literature (Tables 3 and 4), a recent systematic review and meta-analysis article was selected for analysis: Kim et al. (2023) (15), which compiles data from 75 similar studies. Of these studies, 29 were based on imaging examinations and 46 on cadavers. From these, the overall values of the variables under study were taken and considered as reference values. It is important to note that, with regard to the position of the GPF in relation to the maxillary molars, the article in discussion presents a sample size of 36,994 individuals.

The sample size of Kim et al. (2023) [12] allows for the identification of subgroups for certain variables, which are associated with the continent of origin of the studies included. In particular, the results from Europe (where available) were consulted for discussion purposes.

The GPF-PNS, GPF-MMS distances and the anteroposterior diameter of the GPF obtained in this study show no statistically significant differences from the reference values (95% CI). The first two, with mean values that do not intersect the 95% CI of the RVs, suggest the potential for differences, although not proven by the present study.

The GPF-PBHP and GPF-ANS distances are higher than the RVs (95% CI); the GPF-IF distance and the lateromedial diameter of the GPF are lower than the RVs (95% CI).

The GPF-PBHP distance (4.83, 95% CI = 4.60–5.05) is in close proximity to the European value (4.18, 95% CI = 1.83–6.54). Additionally, the GPF-IF distance (35.18, 95% CI = 34.64–35.72) is also in close proximity to the same value (36.79, 95% CI 31.69–41.89).

Conversely, the lateromedial diameter of the GPF (2.24, 95% CI = 2.15–2.34) diverges from the European value in comparison to the overall RV (RV 2.77, 95% CI = 2.58–2.96; European value 3.04, 95% CI = 2.74–3.35).

The position of the GPF in relation to the upper molars is in accordance with the RVs, with no statistically significant differences. Therefore, in the majority of individuals, the GPF is located in opposition to the third molar (77.0%, 95% CI = 68.1–84.4). This result is also consistent with the findings of Bahşi et al. (2019) [2], who reported a prevalence of 66% for the left GPF and 67.33% for the right GPF, and with those of Tomaszewska et al. (2014) [23]. The latter paper also states that European studies do not show heterogeneity in the positioning of the GPF in relation to the upper molars, suggesting that the different results in the literature are explained by the quality of the analysis and the varying methodologies. For this reason, they introduced a classification system, used in this study, with the aim of establishing a generalised method of analysis.

In patients with no remaining teeth, the location of the GPF can be determined by measuring distances to easily identifiable landmarks. Kim et al. (2023) [12] propose that the most reliable landmarks are the MMS, the PBHP and the IF.

The most prevalent form of GPF is the ‘oval/ovoid’, which concurs with the findings of Ortug & Uzel (2019) [17]. No statistically significant differences were observed between the various GPF shapes, with the exception of the “other” category. Although this is a subjective (observational) analysis, the oval shape is the most frequently described in the literature and is consistent with the results obtained in our study.

Although the difference was not statistically significant, the results for the ‘slit’ shape (26%) were higher than the RV (8.4%). This result may be associated with the shorter lateromedial diameter in comparison to the RV (95% CI). Therefore, the findings indicate that the GPFs analysed exhibited a tendency towards greater elongation in the anteroposterior direction in proportion to the lateromedial diameter, in comparison to the RV. Consequently, the prevalence of the round shape (1%) is also lower than that of the RV (9.4%), although this is not statistically significant when the disproportion of the anteroposterior and lateromedial diameters is considered.

The results for the opening direction of the GPF for the oral cavity are not in accordance with the RV, with no vertical or anterior classification recorded. The most prevalent opening direction, despite being the same in relation to the RVs, is significantly higher (95% CI).

It should be noted that the sums of the RVs for the categorical variables (position, shape and opening direction) do not total 100 per cent, as they were estimated individually, considering the studies available for each classification. This is because a statistical analysis of a single global sample was not carried out.

### Comparative morphometric analysis by gender

The metric variables representing the measurements of the male subjects exhibited significantly higher values than those of the female (*p* < 0.05) (Table 5). This finding is consistent with the sexual dimorphism of the skull, which is typically more pronounced in males than in females. These results are consistent with those reported by Gibelli et al. (2017) [8] and Tomaszewska et al. (2014) [23], although these two studies refer to other authors who do not support the findings presented here. Tomaszewska et al. (2014) [23] also suggest that the position of the GPF in relation to reference points (metric variables) could be used to determine gender in the forensic field.

No statistically significant differences were observed in the categorical variables (position, shape and direction of opening) (Table 6). It is thus proposed that the protocol for approaching the GPF in females and males could be the same in terms of position in relation to the upper molars. However, it is recommended that the distances to reference points be taken into account.

### Comparative morphometric analysis by side

There were no statistically significant differences in the variables studied between the right and left sides - Tables 7 and 8. Tomaszewska et al. (2014) [23] recorded differences in the GPF-MMS and GPF-IF variables, and Gibelli et al. (2017) [8] recorded differences in GPF-PNS and GPF-MMS. Therefore, it can be inferred that, clinically, an equivalent protocol can be used when approaching the right and left GPF.

### Implications for clinical practice

With regard to local anaesthesia, the technique for blocking the greater palatine nerve described by Malamed et al. (2017) [15] appears to be suitable for the results obtained. This is based on the following description: ‘*The entrance to the greater palatine foramen should be palpated as a depression or soft zone in the posterior area of the hard palate*.’ It is frequently situated at the midpoint between the gingival margin and the median palatine suture, approximately opposite the distal aspect of the maxillary second molar [15]. Palpation may help locating this anatomical structure especially in cases with atypical shapes and positions of the GPF.

Furthermore, an understanding of the morphometry of the GPF will be beneficial in surgical procedures, with the objective of preventing iatrogenic damage to GPA and GPN [4, 10, 12, 18].

This type of study, to our knowledge, is the first to be carried out in a Portuguese population and makes it possible to adapt medical-dental clinical practice to the target population, based on data with good statistical significance (95% CI).

### Limitations

The inclusion and exclusion criteria present a challenge in selecting the records, as it is necessary to analyse the CBCTs individually. Only a small percentage of the subjects met the requisite criteria, namely having all their upper teeth fully erupted, and including the entire maxilla. In order to select the 50 records included in the sample, 1,256 CBCTs were consulted.

The lack of consistency in the methodologies employed for the morphometric analysis of the GPF precludes any meaningful comparisons of the resulting data. For this reason, this study places particular emphasis on the data and methodologies presented in the systematic reviews conducted by Tomaszewska et al. (2014) [23] and Kim et al. (2023) [12].

The CBCT images were obtained using a sensor with 0,4 mm voxel dimension. Although different voxel sizes may affect detail and diagnostic accuracy, different studies performed on different anatomical structures demonstrate that linear measurements are not necessarily affected [5, 7]. 

This study did not take into account ethnic differences, as this information was not available in the medical records consulted. There is no consensus in the existing literature on whether ethnicity influences the location of the GPF [17]. It is known that there are anatomical differences described between different ethnicities, both in the shape and dimensions of the GPF [13]. Therefore, the applicability of our findings outside the Portuguese population might be limited. However, while Portuguese findings may not fully represent global craniofacial diversity, they can still contribute to a broader understanding of the GPF anatomy.

It is recommended that future studies analysing GPF establish a universal method to ensure the data obtained is truly comparable.

## Conclusion

The most prevalent shape of the GPF is oval (71.0% 95% CI 61.6–79.2). It is located opposite to the maxillary third molar in 77.0% (95% CI 68.1–84.4) of individuals. Its direction of opening into the oral cavity was inferior-anterior-medial in 89.0% (95% CI 81.8–94.0) of cases.

On average, the GPF is located approximately 5 millimetres from the PBHP and 15 millimetres from the MMS. Its anteroposterior diameter is approximately 5 millimetres and its lateromedial diameter is approximately 2 millimetres.

These results provide clinical support for the dentist, preventing iatrogenic damage to the neurovascular bundle and aiding in the anaesthetic technique to block the GPN.

## Data Availability

A dataset was generated and analysed during the study.
